# Supplementary material for: Evaluation of Prebiotics through an In Vitro Gastrointestinal Digestion and Fecal Fermentation Experiment: Further Idea on the Implementation of Machine Learning Technique
Source: Foods. 2022 Aug 17;11(16):2490. doi: 10.3390/foods11162490 (PMC9407061; doi:10.3390/foods11162490)
Supplement: Supplementary file 1 [file foods-11-02490-s001.zip › Supplementary Materials.pptx]

## Slide 1
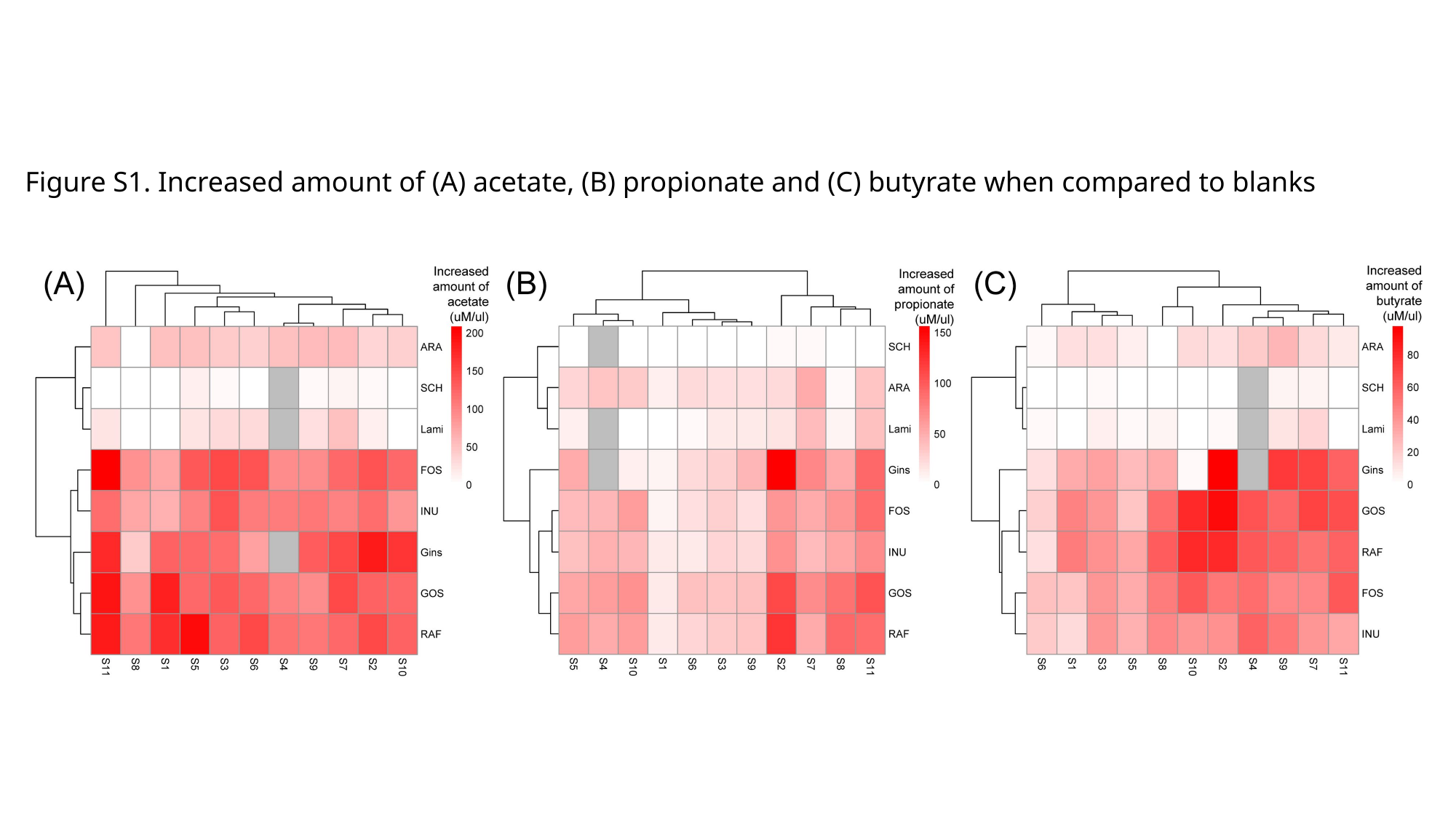

Figure S1. Increased amount of (A) acetate, (B) propionate and (C) butyrate when compared to blanks

## Slide 2
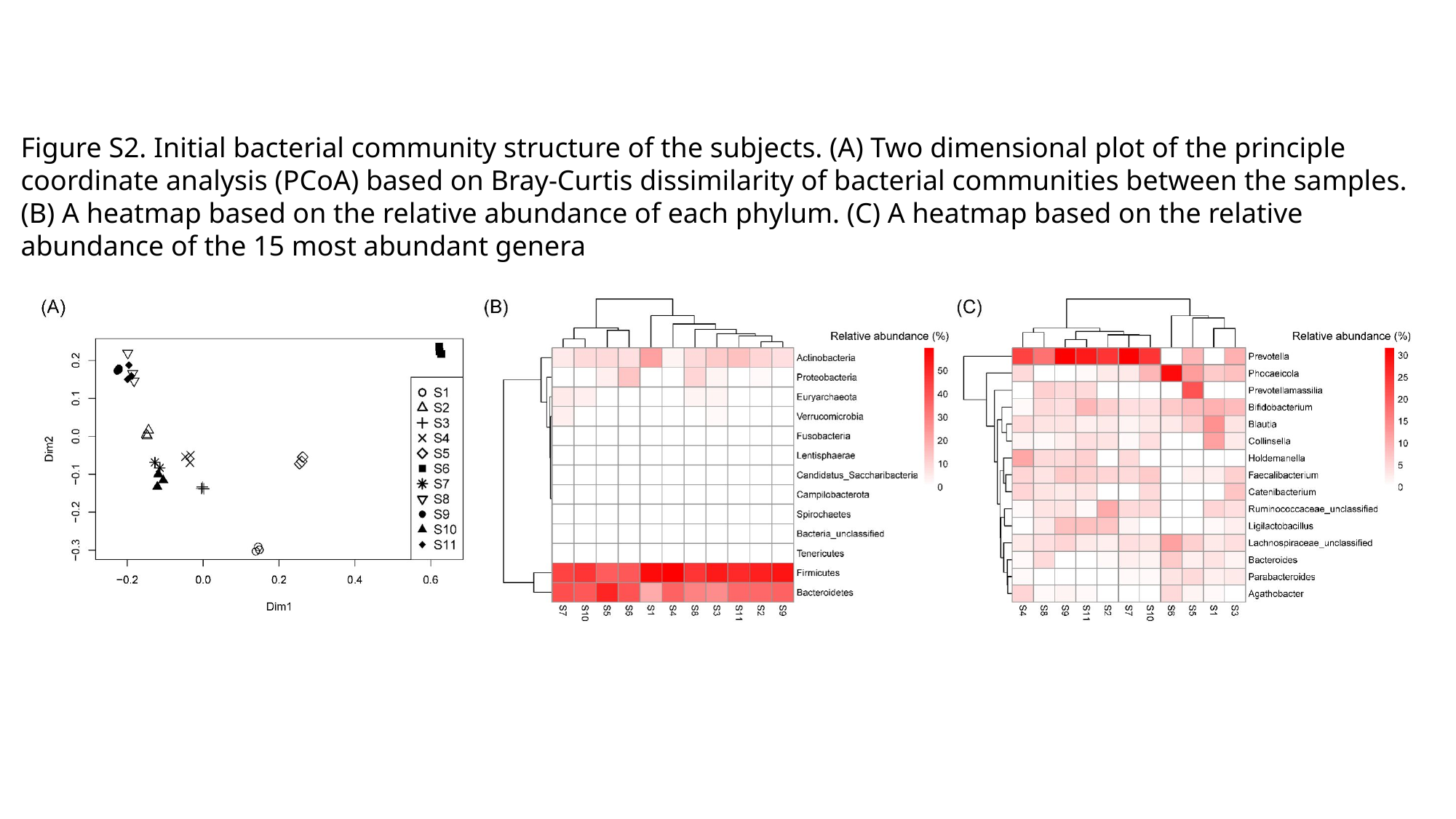

Figure S2. Initial bacterial community structure of the subjects. (A) Two dimensional plot of the principle coordinate analysis (PCoA) based on Bray-Curtis dissimilarity of bacterial communities between the samples. (B) A heatmap based on the relative abundance of each phylum. (C) A heatmap based on the relative abundance of the 15 most abundant genera

## Slide 3
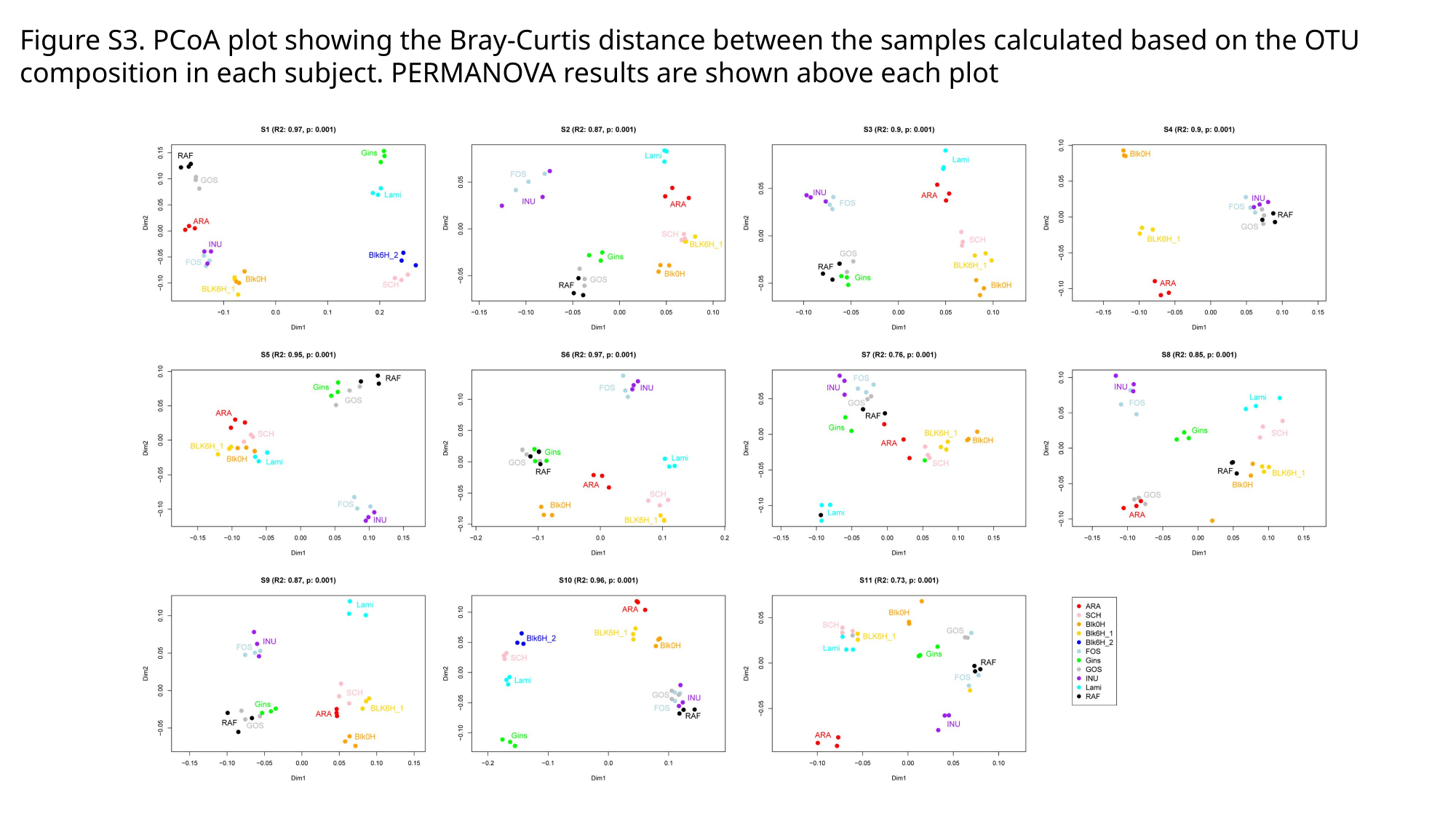

Figure S3. PCoA plot showing the Bray-Curtis distance between the samples calculated based on the OTU composition in each subject. PERMANOVA results are shown above each plot

## Slide 4
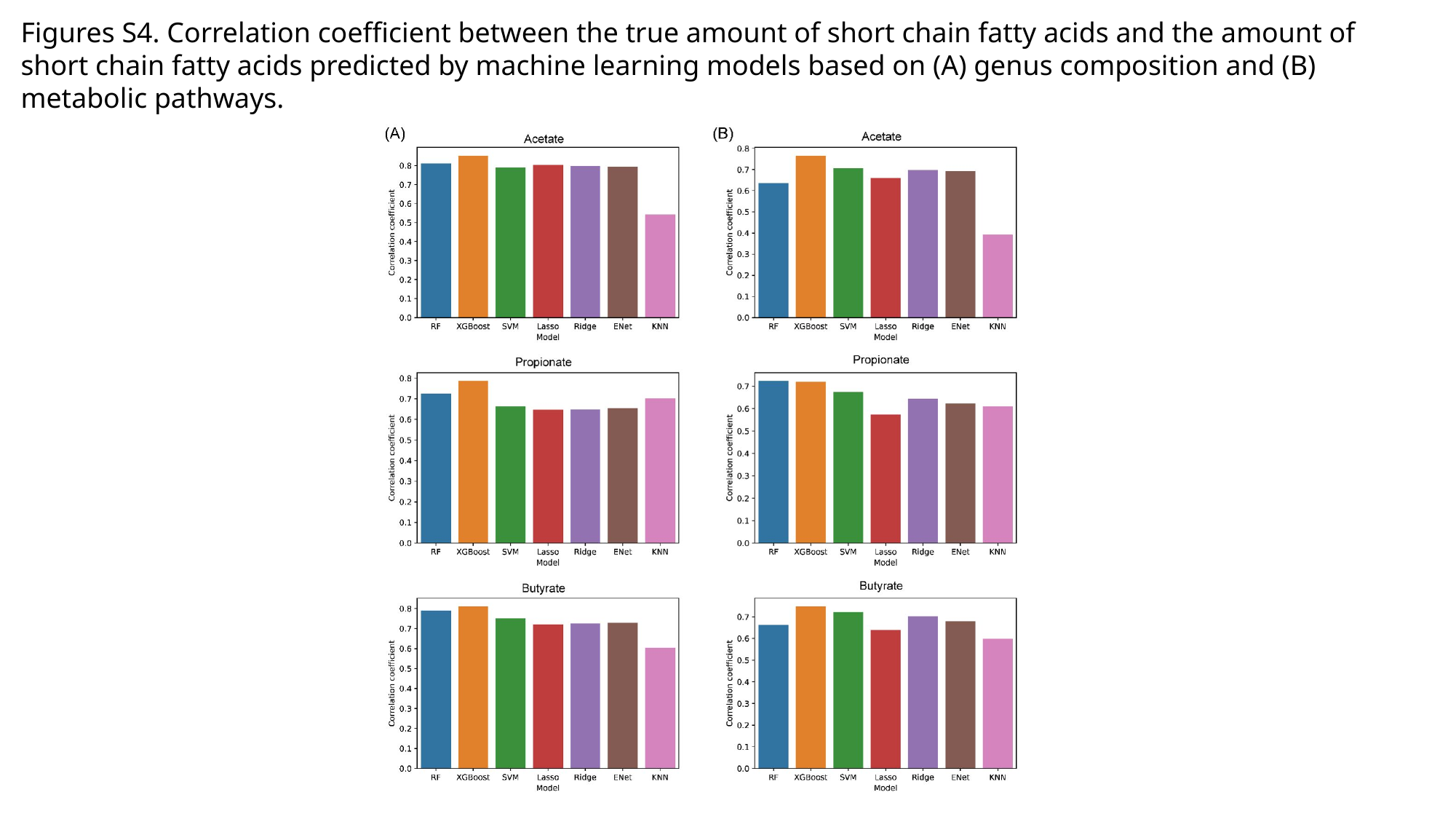

Figures S4. Correlation coefficient between the true amount of short chain fatty acids and the amount of short chain fatty acids predicted by machine learning models based on (A) genus composition and (B) metabolic pathways.
