# Supplementary material for: Evaluation of Prebiotics through an In Vitro Gastrointestinal Digestion and Fecal Fermentation Experiment: Further Idea on the Implementation of Machine Learning Technique
Source: Foods. 2022 Aug 17;11(16):2490. doi: 10.3390/foods11162490 (PMC9407061; doi:10.3390/foods11162490)
Supplement: Supplementary file 1 [file foods-11-02490-s001.zip › supplementary table S1.pdf]

Supplementary Table S1. Best performed hyper-parameters of each machine learning model

Algorithm	Feature	Genus composition			Pathway profile		
	Hyper-parameter	Acetate	Propionate	Butyrate	Acetate	Propionate	Butyrate
Random forest (RF)	bootstrap	False	False	False	False	False	False
	max_depth	10	10	30	30	10	10
	max_features	sqrt	sqrt	sqrt	sqrt	sqrt	sqrt
	min_sample_leaf	2	1	1	2	1	4
	min_samples_split	2	2	2	5	2	2
	n_estimators	100	50	50	50	10	10
Extreme gradient boosting (XGBoost)	colsample_bytree	0.6	0.6	0.6	1	1	1
	gamma	100	0.01	100	1	0.01	0.01
	learning_rate	0.1	0.01	0.01	0.1	0.1	0.01
	max_depth	5	50	5	5	10	5
	min_child_weight	10	2	10	10	5	1
	n_estimators	500	1000	1000	1000	500	1000
	reg_alpha	0.01	100	1	0.01	1	0.01
	reg_lambda	1	0.01	1	1	1	1
	subsample	0.6	0.6	0.6	0.6	1	0.6
Support vector machine (SVM)	C	10000000	100	1000	1000000	10000	1000
	gamma	0.001	100	100	1	100	1000
	kernel	sigmoid	rbf	rbf	rbf	rbf	rbf
Lasso	alpha	0.01	0.01	0.01	0.001	0.001	0.001
Ridge	alpha	0.001	0.01	0.01	0.00001	0.00001	0.00001
Elastic net (ENet)	alpha	0.0001	0.0001	0.0001	0.000001	0.000001	0.000001
	l1_ratio	0.5	0.2	0.2	0.5	0.5	0.5
k-nearest neighbor (KNN)	algorithm	auto	auto	auto	ball_tree	ball_tree	ball_tree
	leaf_size	1	1	1	40	5	5
	metric	minknowski	minknowski	minknowski	jaccard	jaccard	jaccard
	n_neighbors	5	1	20	40	30	30
	p	1	1	1	1	1	1
	weights	distance	uniform	distance	uniform	distance	distance
